# Bodybuilding, dietary supplements and hormones use: behaviour and determinant analysis in young bodybuilders

**DOI:** 10.1186/s13102-021-00378-x

**Published:** 2021-11-24

**Authors:** Paolo Montuori, Ilaria Loperto, Carmine Paolo, Davide Castrianni, Raffaele Nubi, Elvira De Rosa, Raffaele Palladino, Maria Triassi

**Affiliations:** Department of Public Health, University of Naples “Federico II”, Via S. Pansini, 5, 80131 Naples, Italy

**Keywords:** Dietary supplements, Bodybuilding, Athletes, Diet, Gyms

## Abstract

**Background:**

Among athletes, bodybuilders are more predisposed to the use of dietary supplements (DS) and hormones (H) to increase in adaptations to physical training and performance. The purpose of the study was to identify social, psychological, and organisational factors that are associated with the use of food supplements and hormones in young bodybuilders of the metropolitan area of Naples.

**Methods:**

107 athletes, practicing bodybuilding, were consecutively recruited in 30 gyms, randomly selected in the metropolitan area of Naples. Athletes were administered an anonymous questionnaire. The questionnaire consists of 5 sections (socio-demographic, frequency and reasons for bodybuilding, knowledge, attitudes and behaviours). Descriptive statistics were performed using T-test and Chi-square statistics. A score was created for knowledge, attitudes, behaviours. Multivariable logistic regression models were employed to assess association between each score and the use of DS and H. Statistical analyses were carried out using STATA 15.

**Results:**

81.31% of the subjects reported to use DS while 35.51% H. Females are less likely to practise bodybuilding frequently than males (OR 0.18 (95% CI 0.05–0.69), p = 0.01). Subjects who have attended high school or university have a lower probability of taking DS (OR 0.17 (95% CI 0.04–0.65), p = 0.01). H users also use supplements more frequently (OR 61.21 (95% CI 3.99–939.31), p < 0.001). Those who scored higher on knowledge scores are more likely to take DS (OR 1.53 (95% CI 1.11–2.12), p < 0.001). Attitudes are correlated with the use of DS; those who scored higher were less likely to use DS (OR 0.77 (95% CI 0.30–0.98), p = 0.03). People who use DS are 30 times more likely to use H at the same time (OR 30.25 (95% CI 2.51–365.24), p < 0.001). Subjects who have a higher score for knowledge and attitudes are less likely to use H (OR 0.68 (95% CI 0.54–0.87), p < 0.001, OR 0.75 (95% CI 0.62–0.90), p < 0.001).

**Conclusions:**

Prevalence of H and DS’ use, although lower than reported in the literature, is a worrying public health problem. Better knowledge can lead to an informed use. Gym instructors should be trained to provide accurate and scientifically sound information. Health professionals should combine their expertise to provide more comprehensive guidance to the exercisers.

**Supplementary Information:**

The online version contains supplementary material available at 10.1186/s13102-021-00378-x.

## Background

Bodybuilding is a sport in which the athlete is judged on muscular appearance. Proper preparation for a bodybuilding contest generally involves years of strength training followed by a phase in which the athlete focuses on drastically reducing body fat to improve muscle appearance [1, 2].. Bodybuilding’s training programs are characterized by a separation of training into four distinct periods: off-season, pre-contest, peak week, and post-contest. Each period has a specific spectrum of intensity load, total training volume, and exercise type (multi- or single-joint) [3]. The bodybuilders’ goal is to develop muscular hypertrophy and to obtain low fat levels based on a balanced body shape [2]. Since energy restriction prior to competition has a negative effect on anabolic hormones, it can result in a reduction in serum concentrations of hormones such as testosterone, insulin-like growth factor-1 (IGF-1) and insulin [4]. So among athletes, bodybuilders are more predisposed to the use of dietary supplements (DS) and hormones (H) as compared to other sportsmen [4]. The aim of the consumption of these substances is the increase in adaptations to physical training and physical performance. In addition to friends and family, advertisements through media were the main source of information on supplement use; very few obtained information/advice from their physicians [5]. The most common DS currently used are vitamins, minerals, protein powders/liquid, and amino acids [6]. The use of DS is also a risk factor for illicit substance use and may cause so-called inadvertent doping due to the contamination of their ingredients with, for example, oxilofrine, β-methylphenethylamine (BMPEA) and N,β-dimethylphenethylamine (NBDMPEA), the stimulant 4-methylhexan-2-amine (methylhexaneamine, 1,3-dimethylamylamine, DMAA), the anabolic steroids boldione (1,4-androstadiene-3,17-dione) and 5-androstene-3β,17α-diol (17α-AED), the beta-2 agonist higenamine and the beta-blocker bisoprolol [7, 8].

However, the exact health benefits DS are still not well established [9, 10]. Moreover, various possible hazards were described when used inappropriately or without medical consultation [11].

In Italy, a medical prescription is not required for natural health products so DS are easily available for self-care and self-selection.

The main EU rules concerning food supplements is Directive 2002/46/EC [12]. Since an excessive intake can cause undesirable effects [13–17], the directive provides for the establishment of maximum amounts of vitamins and minerals to be added to food supplements. In Italy, in addition to the transposition of these directives (Legislative Decree no. 169 of 21 May 2004 and subsequent amendments [18]), in order to ensure an adequate level of quality and safety of dietary supplements, the Ministry of Health published in November 2018 the “Recommendations on the good manufacturing practices of food supplements”, aimed at providing technical indications that meet the specific needs of the manufacturing industries [19]. At the present time, DS that have as ingredients the substances allowed by the legislation can be placed on the market. If this condition does not occur, it is necessary to request an ad hoc authorization, based solely on the demonstration of safety of use, as governed by EU Regulation 2015/2283 on novel foods [20].

At the same time, it should be emphasized that for the placing on the market of a substance such as DS and/or ingredient of a DS, no proof of efficacy is required.

About 50% of the general population and up to 100% of athletes in some sports have reported taking dietary supplement [11, 21]*.* In a recent study on spanish bodybuilders, 100% of subjects reported consuming DS [4].

Although many mechanisms of action in improving performance have been proposed for DS, there are few studies that demonstrate real efficacy. For example, very high intakes of arginine may increase exercise-induced Human growth hormone (HGH) release, but this does not correspond to an objective increase in sports performance; carnitine should improve skeletal muscle function but there is no evidence that carnitine improves physical performance in healthy subjects [22]. Also, creatine is a popular supplement for elevating energy during short, high-intensity exercises [23]*.*

Several studies have indicated adverse effects of dietary supplements consumption, including cardiovascular, hematological, metabolic, and neurological problems [14, 22]*.* Despite the availability of wide information about supplement utilization from different parts of the world, limited data is available from Italy.

Sometimes bodybuilders use banned substances such as diuretics, stimulants, or anabolic substances, included in the list of prohibited methods and substances published by the World Anti-Doping Agency (WADA) [24]. Previous reports that have analyzed the prevalence of use of anabolic steroids suggest polypharmacy and high doses of injectable agents [25]. Nandrolone decanoate and Sustanon, a combination product of 4 testosterone esters, were the most commonly reportedly used agents [25]. The use of prohibited substances is estimated to be 1–5% in the general population, reaching up to 50% in some sports [26, 27].

The use of doping agents has become a public health problem as it also affects young and non-professional athletes [28]. However, there are objective health risks including cardiovascular illnesses, diabetes, cancer, mental health problems, virilization in women and the suppression of the androgens produced naturally in men [26].

Given that bodybuilders are an high risk category of DS and H consumption and that studies carried out in Italy in this population are lacking, our purpose was to identify the social, psychological, and organisational factors that are associated with the use of food supplements and hormones in young bodybuilders of the metropolitan area of Naples.

## Methods

### Study design and setting

A cross-sectional analysis of data from 107 athletes, practicing bodybuilding, recruited from 30 gyms, randomly selected in the metropolitan area of Naples, has been performed. Inclusion criteria for the gyms were to be located in Naples provinces and have at least one bodybuilding course held by a bodybuilding instructor with a technical card recognized by CONI (Italian National Olympic Committee). For each gym, all subjects enrolled in bodybuilding courses, aged over 18 years and able to understand every item of the questionnaire were eligible, with no regard to sex, skin color, class, or social group. In Italy, the FIPE (Italian weightlifting federation) Federal Council decided to assimilate the contents of resolution no.1568 of 14 February 2017 of the National Council of CONI about the authentic interpretation of the definition of "Activities with overloads and resistances aimed at fitness and physical well-being" specified below: “Physical Culture (bodybuilding) includes all those activities that use any form of load (or resistance) to achieve the goal in terms of sports (competitive and non-competitive), conditioning (fitness) also aimed at competitive performance, well-being (wellness) and recovery of physical efficiency” [29]. For this reason, both professional and non-professional athletes, both competing bodybuilders and amateur bodybuilders have been enrolled. No sample size calculation was performed because data on the total number of athletes practicing bodybuilders in the area was not possible to retrieve. However, our study population size is comparable to study populations from previous studies on the topic conducted in similar settings [4, 30]. Informed consent was obtained from each participant, the project was approved by the Ethics Committee of Medical School of the University “Federico II” and all methods were carried out in accordance with relevant guidelines and regulations.

### Study population and study variables

Athletes were administered an anonymous paper questionnaire. Each participant was instructed on how to fill in the questionnaire, providing a list of the most frequently used DS and H with the commercial name and defining some variables. In particular, it was specified that for the “Reason for Bodybuilding” variable, the answer "Other reasons" included "Improve health", "Lose weight", "Increase strength", "Participate in competitions"; while for the "Bodybuilding Frequency" variable, the "seldomly" response included the subjects who practice less than 3 workouts a week and to the "frequently" group all the others. The questionnaire (see Additional file 1), which was mirrored on existing questionnaires and adapted [31–33], consists of 58 questions focusing on bodybuilding, supplements and hormones. The questionnaire consists of 5 sections. The first Sect. (6 questions) asks socio-demographic information while the second one (5 questions) asks information about frequency and reasons for bodybuilding. The third Sect. (11 questions), concerning knowledge, and the fourth (16 questions), concerning attitudes, were assessed on a three-point Likert scale (agree, uncertain and disagree) while the fifth Sect. (20 questions), concerning behaviours, is based on a 4-degree scale (yes, often, sometimes, never). For each section, using a Likert scale, a score ranging from 1 to 3 or from 1 to 4 was created. For the "knowledge" and "behaviours" sections, the highest score was attributed to the correct answer or to the healthier behaviour, while for the "attitudes" the higher score was attributed to the attitude that could lead to healthier behavior. The questionnaire underwent an internal validation, was pre-tested and modifications were been made to improve validity of responses. Specifically, the questionnaire was administered to 20 public health trainees based at the Department of Public Health to discuss clarity of the questions.

The following information from the survey was included in the following study: age, sex, weight, height, education attainment, employment status, reasons for bodybuilding, frequency of bodybuilding, duration of practice, use of supplements, use of hormones, smoke habits, knowledge, attitude and behaviour’s scores. As regards education attainment, the Italian school system is structured in three cycles of education: primary education, which includes primary school, lasting five years; secondary education, which includes a three-year lower secondary school, and a five-year high school.

### Statistical analysis

Descriptive statistics of the study variables were performed using T-test, Chi-square and Fisher's exact test statistics, as appropriate. For the multivariable analyses, a score was created for each section (knowledge, attitudes, behaviours). Multivariable logistic regression models were employed to assess the association between each score and the use of supplements and hormones.

Four binary outcome variables were selected: bodybuilding frequency (seldomly, frequently), bodybuilding reasons (other reasons, improve body look), use of supplements, use of hormones. Models were adjusted for age, gender, BMI, education attainment, employment, smoking and the remaining outcome variables.

Statistical analyses were carried out using STATA 15.

## Results

Of the 107 participants, 68% are male and 32% are female. 81.31% of the subjects reported to use supplements while 35.51% hormones. Table 1 shows the characteristics of the patients divided by use of supplements, hormones, and by gender.

**Table 1:** Patient characteristics by supplements use, hormone use and sex

	Supplements use					Hormones use					Sex				
	NO		YES		P	NO		YES		P	M		F		p
N (%)	20	(18.69)	87	(81.31)		69	(64.49)	38	(35.51)		73	(68.22)	34	(31.78)	
Sex, n (%)															
Male	16	(80.00)	57	(65.52)	0.210^§^	46	(66.67)	27	(71.05)	0.641^§^					
Female	4	(20.00)	30	(34.48)		23	(33.33)	11	(28.95)						
AGE, mean (SD)	34.45	(10.22)	31.13	(7.40)	0.048*	31.37	(8.65)	32.42	(6.90)	0.738*	32.09	(7.82)	31	(8.62)	0.26*
Education attainment, n (%)															
Up to secondary school	1	(5.00)	24	(27.59)	0.096^§^	14	(20.29)	11	(28.95)	0.449^§^	19	(26.03)	6	(17.65)	**0.045**^**§**^
High-school	14	(70.00)	48	(55.17)		43	(62.32)	19	(50.00)		45	(61.64)	17	(50.00)	
Bachelor degree and above	5	(25.00)	15	(17.24)		12	(17.39)	8	(21.05)		9	(12.33)	11	(32.35)	
Employment status, n (%)															
Unemployed/others	4	(20.00)	28	(32.18)	0.464^§^	20	(28.99)	12	(31.58)	0.926^§^	18	(24.66)	14	(41.18)	0.220^§^
Employed	10	(50.00)	32	(36.78)		28	(40.58)	14	(36.84)		31	(42.47)	11	(32.35)	
Self-employed	6	(30.00)	27	(31.03)		21	(30.43)	12	(31.58)		24	(32.88)	9	(26.47)	
Reason for bodybuilding, n (%)															
Other reason	10	(50.00)	32	(36.78)	0.275^§^	26	(37.68)	16	(42.11)	0.654^§^	26	(53.62)	16	(47.06)	0.259^§^
Improve body look	10	(50.00)	55	(63.22)		43	(62.32)	22	(57.89)		47	(64.38)	18	(52.94)	
Frequency of bodybuilding, n (%)															
Seldomly	3	(15.00)	21	(24.14)	0.377^§^	15	(21.74)	9	(23.68)	0.817^§^	12	(16.44)	12	(35.29)	**0.029**^**§**^
Frequently	17	(85.00)	66	(75.86)		54	(78.26)	29	(76.32)		61	(83.56)	22	(64.71)	
Duration of Bodybuilding, n (%)															
Less than a year	2	(10.00)	10	(11.49)	0.793^§^	8	(11.59)	4	(10.53)	0.350^§^	5	(6.85)	7	(20.59)	0.106^§^
Less than five years	9	(45.00)	45	(51.72)		38	(55.07)	16	(42.11)		38	(52.05)	16	(47.06)	
More than five years	9	(45.00)	32	(36.78)		23	(33.33)	18	(47.37)		30	(41.10)	11	(32.35)	
Hormones, n (%)	1	(5.00)	37	(42.53)	**0.002**^**§**^						27	(36.99)	11	(32.35)	0.641^§^
Supplements, n (%)						50	(72.46)	37	(97.37)	**0.002**^**§**^	57	(78.08)	30	(88.24)	0.210^§^

Statistically significant* p* values (<0.05) are in bold

^*^T Test

^§^Chi square Test/ Fisher's exact Test

81.31% of the athletes declared to use supplements. 65.52% of the subjects who use supplements are male while only the 34.48% are female. The majority of subjects who use supplements (55.17%) completed the high school while the percentages regarding employment status are very similar: 32.18% are unemployed, 36.78% are employed, 31.03% are self-employed. 63.22% of the subjects who use supplements practice bodybuilding for aesthetic reasons, 75.86% declare to practice it frequently while 51.72% declare that they have been training for less than 5 years. For these variables, there is no difference between the group that uses supplements and the one that does not use them (p > 0.05). The average age of those who use supplements is lower than those who do not use them (31.13 vs 34.45, p = 0.048). The 42.53% of subjects who use supplements say they also use hormones (p = 0.002).

35.51% of the interviewed subjects declared to use hormones. Of these, 71.05% are male and only 28.05% are female. The average age of hormone users is 32.42 and there is no difference with that of non-users (31.37, p = ns). As for supplements, even in the case of hormones, those who use them have more frequently a high school diploma (50.00%), while as regards employment status, the distribution is very similar in the three groups (unemployed: 31.58%; employed: 36.84%; self-employed: 31.58%, p = ns). Most people who use hormones practice bodybuilding to improve body look (57.89%), 76.32% train frequently and 47.37% declare to practice bodybuilding for more than 5 years.

For the use of hormones, there is no difference in the two groups for any variable (p > 0.05).

It is interesting to note that those who use hormones also tend to use supplements at the same time (97.37%, p = 0.002).

On average, females have a higher level of education than males (p = 0.045), while males tend to train more frequently (p = 0.029). For the other variables considered, there is no statistically significant difference.

As shown in Table 2, female subjects are less likely to practise bodybuilding frequently than male subjects (Fully adjusted model—OR 0.18 (95% CI 0.05–0.69), p = 0.01). For the "bodybuilding frequency" outcome variable, no other statistically significant association was found.

**Table 2:** Association between knowledge, attitudes, and behaviour and bodybuilding frequency (X, Y)

	Partially adjusted model 1				Partially adjusted model 2				Partially adjusted model 3				Partially adjusted model 4				Fully adjusted model			
	OR	95% CI		p	OR	95% CI		p	OR	95% CI		p	OR	95% CI		p	OR	95% CI		p
Age	**1.08**	**1.01**	**1.17**	**0.04**	1.07	0.99	1.15	0.06	1.07	0.99	1.15	0.07	1.07	1.00	1.16	0.06	1.07	0.99	1.15	0.09
Sex (F)	**0.22**	**0.07**	**0.95**	**0.04**	**0.23**	**0.07**	**0.80**	**0.02**	**0.17**	**0.05**	**0.65**	**0.01**	**0.22**	**0.06**	**0.75**	**0.02**	**0.18**	**0.05**	**0.68**	**0.01**
BMI	0.88	0.71	1.08	0.22	0.85	0.70	1.05	0.13	0.82	0.66	1.01	0.07	0.85	0.69	1.04	0.12	0.82	0.66	1.02	0.08
Education attainment	1.13	0.52	2.46	0.32	1.11	0.51	2.43	0.79	1.28	0.57	2.84	0.60	1.14	0.52	2.47	0.74	1.24	0.55	2.78	0.61
Employment status	1.15	0.61	2.17	0.42	1.10	0.58	2.11	0.77	1.18	0.62	2.27	0.61	1.14	0.60	2.17	0.68	1.15	0.59	2.24	0.68
Smoke	0.81	0.43	1.49	0.49	0.80	0.43	1.51	0.50	0.81	0.43	1.51	0.50	0.80	0.43	1.49	0.49	0.80	0.42	1.52	0.50
Supplements’ use	0.82	0.18	3.61	0.79	0.76	0.17	3.42	0.72	0.65	0.14	3.01	0.58	0.83	0.19	3.66	0.80	0.57	0.12	2.71	0.48
Hormones’ use	0.92	0.31	2.70	0.90	1.06	0.32	3.44	0.92	0.59	0.18	1.94	0.38	0.91	0.30	2.74	0.87	0.70	0.19	2.52	0.58
Knowledge					1.06	0.87	1.29	0.55									1.08	0.88	1.32	0.48
Attitudes									0.86	0.73	1.01	0.06					0.85	0.72	1.01	0.05
Behaviours													1.00	0.92	1.08	0.95	1.02	0.93	1.11	0.70

Statistically significant* p* values (<0.05) are in bold

Estimates were derived from multivariable logistic regression models adjusted for age, gender, BMI, education attainment, employment, smoking and the remaining outcome variables

For the "bodybuilding reason" outcome variable, no variable considered was associated in any of the constructed models (Table 3).

**Table 3:** Association between knowledge, attitudes, and behaviour and bodybuilding reasons (X, Y)

	Partially adjusted model 1				Partially adjusted model 2				Partially adjusted model 3				Partially adjusted model 4				Fully adjusted model			
	OR	95% CI		p	OR	95% CI		p	OR	95% CI		p	OR	95% CI		p	OR	95% CI		p
Age	0.97	0.93	1.03	0.38	0.97	0.92	1.03	0.38	0.97	0.92	1.03	0.44	0.97	0.93	1.03	0.40	0.97	0.92	1.03	0.46
Sex (F)	0.48	0.17	1.36	0.17	0.48	0.17	1.36	0.17	0.50	0.18	1.41	0.19	0.47	0.17	1.33	0.15	0.48	0.16	1.39	0.18
BMI	0.94	0.80	1.11	0.49	0.94	0.80	1.11	0.49	0.95	0.81	1.12	0.54	0.93	0.80	1.10	0.45	0.94	0.80	1.11	0.50
Education attainment	0.93	0.49	1.79	0.66	0.93	0.48	1.79	0.83	0.91	0.47	1.75	0.78	0.91	0.47	1.76	0.78	0.90	0.47	1.75	0.76
Employment status	1.32	0.77	2.26	0.31	1.32	0.76	2.28	0.32	1.30	0.76	2.23	0.33	1.34	0.78	2.30	0.29	1.32	0.76	2.31	0.32
Smoke	1.39	0.82	2.35	0.22	1.39	0.82	2.35	0.22	1.40	0.82	2.37	0.22	1.40	0.83	2.38	0.21	1.41	0.83	2.39	0.21
Supplements’ use	1.98	0.64	6.11	0.24	1.97	0.61	6.32	0.25	2.11	0.67	6.66	0.20	1.89	0.60	5.91	0.27	2.06	0.62	6.90	0.24
Hormones’ use	0.75	0.30	1.85	0.53	0.75	0.28	2.06	0.58	0.88	0.32	2.40	0.81	0.79	0.31	2.01	0.62	0.87	0.30	2.57	0.81
Knowledge					1.00	0.85	1.17	0.98									0.99	0.84	1.17	0.90
Attitudes									1.05	0.92	1.19	0.45					1.04	0.91	1.19	0.54
Behaviours													1.02	0.95	1.09	0.56	1.01	0.94	1.09	0.69

Statistically significant* p* values (<0.05) are in bold

Estimates were derived from multivariable logistic regression models adjusted for age, gender, BMI, education attainment, employment, smoking and the remaining outcome variables

As shown in Table 4, compared to those with a middle school diploma, subjects who have attended high school or university have a lower probability of taking supplements (Fully adjusted model—OR 0.17 (95% CI 0.04–0.65), p = 0.01). Hormone users also use supplements more frequently (Fully adjusted model—OR 61.21 (95% CI 3.99–939.31), p < 0.001). In addition, those who scored higher on knowledge scores are more likely to take supplements (Fully adjusted model—OR 1.53 (95% CI 1.11–2.12), p < 0.001). Only in the fully adjusted model, attitudes are correlated with the use of supplements; those who scored higher were less likely to use supplements (Fully adjusted model—OR 0.77 (95% CI 0.30–0.98), p = 0.03).

**Table 4:** Association between knowledge, attitudes, and behaviour and use of supplements (X, Y)

	Partially adjusted model 1				Partially adjusted model 2				Partially adjusted model 3				Partially adjusted model 4				Fully adjusted model			
	OR	95% CI		p	OR	95% CI		p	OR	95% CI		p	OR	95% CI		p	OR	95% CI		p
Age	0.94	0.88	1.01	0.09	**0.92**	**0.85**	**0.99**	**0.03**	0.94	0.87	1.00	0.06	0.95	0.89	1.02	0.19	0.92	0.84	0.99	0.05
Sex (F)	3.22	0.55	18.93	0.19	4.80	0.79	29.05	0.09	2.65	0.42	16.78	0.30	2.60	0.43	15.62	0.30	2.60	0.39	17.29	0.32
BMI	0.91	0.72	1.15	0.43	0.97	0.76	1.23	0.79	0.92	0.72	1.17	0.49	0.86	0.68	1.10	0.24	0.94	0.73	1.23	0.69
Education Attainment	**0.28**	**0.10**	**0.84**	**0.02**	**0.19**	**0.05**	**0.67**	**0.01**	**0.30**	**0.10**	**0.92**	**0.04**	**0.23**	**0.07**	**0.75**	**0.01**	**0.17**	**0.04**	**0.65**	**0.01**
Employment Status	1.07	0.49	2.35	0.86	0.89	0.39	2.04	0.79	1.18	0.52	2.68	0.69	1.07	0.47	2.43	0.87	1.08	0.43	2.67	0.83
Smoke	1.23	0.56	2.71	0.61	1.51	0.61	3.75	0.37	1.29	0.58	2.88	0.53	1.25	0.56	2.78	0.59	1.86	0.65	4.99	0.22
Frequencies	0.83	0.17	3.95	0.82	0.84	0.16	4.40	0.84	0.60	0.11	3.16	0.55	0.95	0.20	4.44	0.94	0.61	0.11	3.38	0.57
Hormones’ use	**23.02**	**2.63**	**201.57**	**0.00**	**87.93**	**6.32**	**1223.7**	**0.00**	**13.52**	**1.42**	**128.33**	**0.02**	**33.12**	**3.29**	**333.60**	**0.00**	**61.21**	**3.99**	**939.31**	**0.00**
Knowledge					**1.47**	**1.12**	**1.93**	**0.00**									**1.53**	**1.11**	**2.12**	**0.00**
Attitudes									0.85	0.69	1.05	0.14					**0.77**	**0.60**	**0.98**	**0.03**
Behaviours													1.09	0.98	1.22	0.09	1.11	0.98	1.25	0.07

Statistically significant* p* values (<0.05) are in bold

Estimates were derived from multivariable logistic regression models adjusted for age, gender, BMI, education attainment, employment, smoking and the remaining outcome variables

Finally, Table 5 shows the associations between hormone intake and the other variables taken into consideration. People who use supplements are 30 times more likely to use hormones at the same time (Fully adjusted model—OR 30.25 (95% CI 2.51–365.24), p < 0.001). Subjects who have a higher score for knowledge and attitudes are less likely to use hormones (Fully adjusted model—OR 0.68 (95% CI 0.54–0.87), p < 0.001, OR 0.75 (95% CI 0.62–0.90), p < 0.001).

**Table 5:** Association between knowledge, attitudes, and behaviour and use of hormones (X, Y)

	Partially adjusted model 1				Partially adjusted model 2				Partially adjusted model 3				Partially adjusted model 4				Fully adjusted model			
	OR	95% CI		p	OR	95% CI		p	OR	95% CI		p	OR	95% CI		p	OR	95% CI		p
Age	1.03	0.97	1.10	0.26	1.05	0.98	1.31	0.13	1.03	0.96	1.09	0.41	1.03	0.97	1.10	0.29	1.04	0.97	1.11	0.26
Sex (F)	1.03	0.34	3.14	0.95	0.67	0.19	2.40	0.54	0.73	0.21	2.57	0.62	1.12	0.36	3.46	0.85	0.47	0.12	1.93	0.30
BMI	1.18	0.99	1.40	0.06	1.16	0.95	1.42	0.15	1.11	0.90	1.36	0.32	1.19	1.00	1.42	0.05	1.08	0.86	1.36	0.49
Education attainment	1.11	0.56	1.20	0.76	1.38	0.64	2.95	0.41	1.36	0.63	2.94	0.42	1.25	0.61	2.56	0.54	1.56	0.68	3.59	0.30
Employment status	0.88	0.50	1.56	0.67	1.24	0.63	2.42	0.53	1.02	0.54	1.92	0.95	0.86	0.48	1.53	0.61	1.27	0.62	2.61	0.51
Smoke	0.79	0.45	1.39	0.42	0.99	0.52	1.88	0.98	0.77	0.40	1.45	0.41	0.78	0.43	1.38	0.39	0.94	0.47	1.88	0.86
Frequencies	0.86	0.29	2.48	0.78	1.10	0.31	3.89	0.89	0.57	0.17	1.95	0.37	0.90	0.29	2.73	0.85	0.72	0.17	2.96	0.65
Supplements’ use	**21.07**	**2.47**	**179.66**	**0.00**	**47.04**	**4.78**	**463.20**	**0.00**	**11.21**	**1.24**	**101.27**	**0.03**	**24.29**	**2.80**	**210.70**	**0.00**	**30.25**	**2.51**	**365.24**	**0.00**
Knowledge					**0.65**	**0.52**	**0.81**	**0.00**									**0.68**	**0.54**	**0.87**	**0.00**
Attitudes									**0.71**	**0.60**	**0.84**	**0.00**					**0.75**	**0.62**	**0.90**	**0.00**
Behaviours													0.93	0.86	1.00	0.05	1.00	0.91	1.09	0.99

Statistically significant* p* values (<0.05) are in bold

Estimates were derived from multivariable logistic regression models adjusted for age, gender, BMI, education attainment, employment, smoking and the remaining outcome variables

As far as the source of information is concerned, it is interesting to note that for DS 83.18% of subjects declare to inquire at the gym, 71.03% on the internet and 64.49% from their doctor/nutritionist. For H, on the other hand, 42.45% of subjects declare to obtain information in the gym, 25.23% on the internet, 38.22% from their doctor/nutritionist (data not shown).

## Discussion

The hypotheses tested in the present research concern the possibility that the basic knowledge of bodybuilders influences the use of DS and H. If this is true in professional bodybuilders, it is interesting to know whether in a mixed population of professional and non-professional bodybuilders, this phenomenon occurs. If this were the case, in fact, the phenomenon could be conditioned with health education interventions.

In the present study, 81.31% of the subjects reported to use supplements while 35.51% hormones. Subjects who have attended high school or university have a lower probability of taking supplements, while hormone users also use supplements more frequently. In addition, those who scored higher on knowledge scores are more likely to take supplements; instead, those who scored higher on attitude scores were less likely to use supplements.

Finally, people who use supplements are 30 times more likely to use hormones at the same time and subjects who have a higher score for knowledge and attitudes are less likely to use hormones.

Gym trainees constitute an important target for dietary supplement markets and professional bodybuilders frequently consume banned substances and hormones in large dosages [4, 33, 34]. The chosen population, therefore, being made up of professional and non-professional bodybuilders, should place itself between the two populations indicated above. Nevertheless, the prevalence of dietary supplements use we found (81.31%) is higher than described in most recent studies in gym users (36.8–43.8%) [5, 34–36], but it is closer to what is reported by Morrison et al. (2004) in USA gym trainees (84.7%) [37]. It is interesting to note that in international athletes and professional bodybuilders the prevalence of the use of supplements is 82.2% and 100%, respectively [4, 32]. The prevalence of steroid hormones’ use among bodybuilders reported in the literature is between 83.3 and 72.9% [4, 25]. In the present study, only 35.51% of subjects report using hormones. This discrepancy would be attributable to the belonging of the subjects to the non-professional category.

However, the discrepancies in the reported prevalence rates of supplements and hormones use may be related to sociodemographic and cultural characteristics, the type of gyms included or methodologic aspects, namely what was considered to be a supplement or hormone and the method of data acquisition. However, research indicates that direct questioning of sensitive information, such as the use of performance enhancing drugs, is characterized by underreporting [28]. We found that supplement and hormones consumption was more prevalent among men, although not statistically significant. Even though the role of gender as a determinant of use is not clearly established, our result is consistent with previous studies [5, 35, 38, 39].

The associated use of supplements and hormones is consistent with what is already present in literature, confirming the trend towards polypharmacotherapy [25].

To our knowledge, the scoring method used for analysing questionnaires has never been used to investigate the relationships between use of supplements and hormones and risk factors. Moreover, in literature there are several articles that associate the frequency or reasons of exercise with the use of substances, characterizing them as factors of considerable influence in the athlete's decision-making process [36, 40]. Therefore, we decided to choose 4 outcome variables that characterized the entire decision-making process of the athletes to evaluate which associations were with modifiable and non-modifiable factors. In particular, from the multinomial logistic analysis, it emerges that female subjects tend to practice bodybuilding less frequently. This attitude is likely to reflect specific cultural characteristics. Gwizdek et al. reported that gender did not have any significant influence on exercise dependence [41].

The data of considerable interest that emerged from multivariable models is the role of knowledge in the choice of the substances used. It seems, in fact, that subjects with higher education attainment, and with a higher score in the knowledge section, are able to discriminate between supplements and hormones, know the differences and seem able to choose the substance that harms them less to health. Alhomoud et al. suggest that there are significant differences in the knowledge of health sciences and non-health sciences students pertaining to the health benefits and safety of these supplements, what they are and the source of information and help which should be sought when using them [33]. To the best of our knowledge, there are no specific studies conducted on the population of amateur bodybuilders.

The present study is one of the few that investigates the use of supplements and hormones among amateur bodybuilders by integrating the data with that of ad hoc scores on knowledge, attitudes and behaviours. This approach allows us to understand which factors can be used to change behaviours and gain health. The present study, however, has some limitations. First of all, a complete list of substances considered supplements or hormones has not been provided and the result could be strongly influenced by the subject's personal culture. Hence, the ability to act without conditioning may have overestimated the use of supplements and underestimated the use of hormones. The results obtained, however, compared with literature, lead us to believe that they are not too far from reality. Secondly, the main source of information was not requested in a specific question. However, 6 questions in the behavior section concern the main sources of information (gym, internet, doctor/nutritionist) and can offer an overview, albeit not exhaustive, of the issue. In retrospect, we realized that it could be useful to know where the correct information was coming from. In addition, a question on whether use was based on medical advice or not was not included in the tool and it is difficult to generalize the results because the study was carried out in province of Naples only. Finally, we suspect the variable scoring system likely did not capture all differences to be identified, although, to better identify the role of the single scores and their association with the other outcome variables, it was decided to use partially adjusted models and a fully adjusted model.

## Conclusions

The high prevalence of dietary supplement use among Naples bodybuilders reflects a public health concern as, although there are supplements that appear to be effective, indiscriminate use can be correlated with serious health risks. The prevalence of hormone use, although lower than reported in the literature, is similarly a worrying public health problem. The two problems appear related as evidenced by frequent combined use. Better knowledge can lead to an informed use of these substances. It is desirable that gym instructors and coaches, who are the people who are most likely to interface with such athletes, should be sufficiently trained to be able to provide accurate and scientifically sound information on dietary supplements and hormones to the gym users.

Health professionals, including physicians, dietitians, and pharmacists, should combine their expertise with that of coaches and athletic trainers in order to provide more comprehensive guidance to the exercisers. Specific and multidisciplinary health education programs could be created involving prevention departments and local health authorities.

## Supplementary Information

**Additional file 1.** Anonymous questionnaire on knowledge, attitudes and behaviors regarding the use of food supplements in body builders. The questionnaire mirrored on existing questionnaires and adapted, consists of questions focusing on bodybuilding, supplements and hormones.

## Data Availability

The questionnaire used during the current study is included in this published article and its supplementary information files.

## Abbreviations

DS: Dietary supplements
H: Hormones
N: Number
SD: Standard deviation
F: Female
M: Male
BMI: Body mass index

## References

[1] Rossow LM, Fukuda DH, Fahs CA, Loenneke JP, Stout JR (2013). Natural bodybuilding competition preparation and recovery: a 12-month case study. Int J Sports Physiol Perform.

[2] Lambert CP, Frank LL, Evans WJ (2004). Macronutrient considerations for the sport of bodybuilding. Sports Med.

[3] Alves RC, Prestes J, Enes A, de Moraes WMA, Trindade TB, de Salles BF, et al. Training programs designed for muscle hypertrophy in bodybuilders: a narrative review. Sports (Basel). 2020;8. 10.3390/sports8110149. 10.3390/sports8110149 PMC7698840 33218168

[4] Sánchez-Oliver AJ, Grimaldi-Puyana M, Domínguez R. Evaluation and behavior of Spanish bodybuilders: doping and sports supplements. Biomolecules. 2019;9. 10.3390/biom9040122. 10.3390/biom9040122 PMC6523090 30925786

[5] Attlee A, Haider A, Hassan A, Alzamil N, Hashim M, Obaid RS (2018). Dietary supplement intake and associated factors among gym users in a university community. J Diet Suppl.

[6] Spendlove J, Mitchell L, Gifford J, Hackett D, Slater G, Cobley S (2015). Dietary intake of competitive bodybuilders. Sports Med.

[7] Sundgot-Borgen J, Berglund B, Torstveit MK (2003). Nutritional supplements in Norwegian elite athletes–impact of international ranking and advisors. Scand J Med Sci Sports.

[8] Duiven E, van Loon LJC, Spruijt L, Koert W, de Hon OM. Undeclared doping substances are highly prevalent in commercial sports nutrition supplements. J Sports Sci Med. 2021:328–38. 10.52082/jssm.2021.328 PMC8219275 34211326

[9] Huang SH, Johnson K, Pipe AL (2006). The use of dietary supplements and medications by Canadian athletes at the Atlanta and Sydney Olympic Games. Clin J Sport Med.

[10] Lukaski HC (2004). Vitamin and mineral status: effects on physical performance. Nutrition.

[11] Schwenk TL, Costley CD (2002). When food becomes a drug: nonanabolic nutritional supplement use in athletes. Am J Sports Med.

[12] European Parliament C of the EU. Directive 2002/46/EC of the European Parliament and of the Council of 10 June 2002 on the approximation of the laws of the Member States relating to food supplements. 2002:0051–7. https://eur-lex.europa.eu/legal-content/EN/TXT/PDF/?uri=CELEX:32002L0046&from=EN.

[13] Tavani A, Colombo P, Scarpino V, Zuccaro P, Pacifici R, La Vecchia C. A survey of dietary supplement use among Italian sporting club athletes.

[14] Palmer ME, Haller C, McKinney PE, Klein-Schwartz W, Tschirgi A, Smolinske SC (2003). Adverse events associated with dietary supplements: an observational study. Lancet.

[15] Klontz KC, DeBeck HJ, LeBlanc P, Mogen KM, Wolpert BJ, Sabo JL (2015). The role of adverse event reporting in the FDA response to a multistate outbreak of liver disease associated with a dietary supplement. Public Health Rep.

[16] Cohen PA (2014). Hazards of hindsight–monitoring the safety of nutritional supplements. N Engl J Med.

[17] Geller AI, Shehab N, Weidle NJ, Lovegrove MC, Wolpert BJ, Timbo BB (2015). Emergency department visits for adverse events related to dietary supplements. N Engl J Med.

[18] Repubblica_Italiana. DECRETO LEGISLATIVO 21 maggio 2004, n. 169. Attuazione della direttiva 2002/46/CE relativa agli integratori alimentari. 2004.

[19] Salute M della. Norme di buona fabbricazione di Integratori alimentari. 2018. http://www.salute.gov.it/imgs/C_17_pagineAree_3139_listaFile_itemName_0_file.pdf.

[20] European Parliament and the Council of the European Union. Regulation (EU) 2015/2283 of the European Parliament and of the Council of 25 November 2015 on novel foods, amending Regulation (EU) No 1169/2011 of the European Parliament and of the Council and repealing Regulation (EC) No 258/97 of the European Parliament and of the Council and Commission Regulation (EC) No 1852/2001. https://www.fsai.ie/uploadedFiles/Legislation/Food_Legisation_Links/Novel_Foods_And_Ingredients/Reg2015_2283.pdf.

[21] Tokish JM, Kocher MS, Hawkins RJ (2004). Ergogenic aids: a review of basic science, performance, side effects, and status in sports. Am J Sports Med.

[22] Guyda HJ (2005). Use of dietary supplements and hormones in adolescents: a cautionary tale. Paediatr Child Health.

[23] van Loon LJ, Oosterlaar AM, Hartgens F, Hesselink MK, Snow RJ, Wagenmakers AJ (2003). Effects of creatine loading and prolonged creatine supplementation on body composition, fuel selection, sprint and endurance performance in humans. Clin Sci (Lond).

[24] WADA. International Standard Prohibited LisT 2021. 2021. https://www.wada-ama.org/sites/default/files/resources/files/2021list_en.pdf.

[25] Perry PJ, Lund BC, Deninger MJ, Kutscher EC, Schneider J (2005). Anabolic steroid use in weightlifters and bodybuilders: an internet survey of drug utilization. Clin J Sport Med.

[26] Bird SR, Goebel C, Burke LM, Greaves RF (2016). Doping in sport and exercise: anabolic, ergogenic, health and clinical issues. Ann Clin Biochem.

[27] de Hon O, Kuipers H, van Bottenburg M (2015). Prevalence of doping use in elite sports: a review of numbers and methods. Sports Med.

[28] Stubbe JH, Chorus AM, Frank LE, de Hon O, van der Heijden PG (2014). Prevalence of use of performance enhancing drugs by fitness centre members. Drug Test Anal.

[29] CONI. Deliberazione del Consiglio Nazionale n. 1568 del 14 Feb 2017. 2017.

[30] Tian HH, Ong WS, Tan CL (2009). Nutritional supplement use among university athletes in Singapore. Singapore Med J.

[31] Villanova Colmenero M, Martínez-Sanz JM, Norte Navarro A, Ortíz-Moncada R, Hurtado JA, Baladia E (2015). Variables used in questionnaires about ergonutritionals supplements intake. Nutr Hosp.

[32] Jovanov P, Đorđić V, Obradović B, Barak O, Pezo L, Marić A (2019). Prevalence, knowledge and attitudes towards using sports supplements among young athletes. J Int Soc Sports Nutr.

[33] Alhomoud FK, Basil M, Bondarev A. Knowledge, attitudes and practices (KAP) relating to dietary supplements among health sciences and non-health sciences students in one of the universities of United Arab Emirates (UAE). J Clin Diagn Res. 2016;10:JC05–9. 10.7860/JCDR/2016/19300.8439 PMC5071968 27790468

[34] Ruano J, Teixeira VH (2020). Prevalence of dietary supplement use by gym members in Portugal and associated factors. J Int Soc Sports Nutr.

[35] Abo Ali EA, Elgamal HH (2016). Use of dietary supplements among gym trainees in Tanta city. Egypt J Egypt Public Health Assoc.

[36] Goston JL, Correia MI (2010). Intake of nutritional supplements among people exercising in gyms and influencing factors. Nutrition.

[37] Morrison LJ, Gizis F, Shorter B (2004). Prevalent use of dietary supplements among people who exercise at a commercial gym. Int J Sport Nutr Exerc Metab.

[38] Sousa M, Fernandes MJ, Carvalho P, Soares J, Moreira P, Teixeira VH (2016). Nutritional supplements use in high-performance athletes is related with lower nutritional inadequacy from food. J Sport Health Sci.

[39] Tsitsimpikou C, Chrisostomou N, Papalexis P, Tsarouhas K, Tsatsakis A, Jamurtas A (2011). The use of nutritional supplements among recreational athletes in Athens, Greece. Int J Sport Nutr Exerc Metab.

[40] Conner M, Kirk SF, Cade JE, Barrett JH (2003). Environmental influences: factors influencing a woman’s decision to use dietary supplements. J Nutr.

[41] Gwizdek K, Brzęk A, Bąk-Sosnowska M, Dittfeld A, Knapik A, Ziaja D (2018). The use of steroids by gym athletes: an attempt to diagnose the problem scale and possible causes. J Sports Med Phys Fitness.

