# Supplementary material for: Bodybuilding, dietary supplements and hormones use: behaviour and determinant analysis in young bodybuilders
Source: BMC Sports Sci Med Rehabil. 2021 Nov 24;13:147. doi: 10.1186/s13102-021-00378-x (PMC8613966; doi:10.1186/s13102-021-00378-x)
Supplement: Supplementary file 1 — Additional file 1. Anonymous questionnaire on knowledge, attitudes and behaviors regarding the use of food supplements in body builders. The questionnaire mirrored on existing questionnaires and adapted, consists of questions focusing on bodybuilding, supplements and hormones. [file 13102_2021_378_MOESM1_ESM.docx]

**Additional file 1**

**BODYBUILDING, DIETARY SUPPLEMENTS AND HORMONES USE: BEHAVIOUR AND DETERMINANT ANALYSIS IN YOUNG BODYBUILDERS**

Paolo Montuori, Ilaria Loperto, Carmine Paolo, Davide Castrianni, Raffaele Nubi, Elvira De Rosa, Raffaele Palladino, Maria Triassi.

“ANONYMOUS QUESTIONNAIRE ON KNOWLEDGE, ATTITUDES AND BEHAVIORS REGARDING THE USE OF FOOD SUPPLEMENTS IN BODY BUILDERS”

Conflicting evaluations divide public opinion between the risks and benefits of using supplementary substances to the diet to improve athletic performance. In this regard, the Department of Public Health of the University of Naples "Federico II" has undertaken a survey with the aim of evaluating the knowledge, attitudes and behaviors regarding the use of Food Supplements and Hormones by individuals who practice bodybuilding in a metropolitan area.

**Age _________ Gender**  **M**  **F Wheight_________kg Height________cm**

**Education Attainment:** up to secondary school high-school  bachelor degree and above

**Employment Status:** Unemployed/Others  Employed  Self-employed 

**Reason for bodybuilding:** Other reasons  Improve body look 

**Frequency of Bodybuilding:** Seldomly  Frequently 

**Duration of Bodybuilding:** Less than a year  Less than five years  More than five years 

**Use of Food Supplements:** Yes  No 

**Use if Hormones:** Yes  No 

| **KNOWLEDGE** | | **Agree** | **Unsure** | **Disagree** |  |
| --- | --- | --- | --- | --- | --- |
| 1 | A proper nutrition includes vitamins, amino acids and minerals. |  |  |  |  |
| 2 | Vitamins, amino acids and minerals are Food Supplements. |  |  |  |  |
| 3 | Food Supplements are drugs. |  |  |  |  |
| 4 | Anabolic steroids are Food Supplements. |  |  |  |  |
| 5 | Anabolic steroids are hormones. |  |  |  |  |
| 6 | A proper nutrition includes hormones. |  |  |  |  |
| 7 | Hormones are food supplements. |  |  |  |  |
| 8 | Hormones are drugs. |  |  |  |  |
| 9 | Hormones have to be prescribed only by the doctor. |  |  |  |  |
| 10 | In Italy there is a law that regulates Food Supplements. |  |  |  |  |
| 11 | In Italy, the national law indicates that Food Supplements must have a label the maximum daily dose. |  |  |  |  |
| **ATTITUDES** | | **Agree** | **Unsure** | **Disagree** |  |
| 12 | Bodybuilding improves the health. |  |  |  |  |
| 13 | Bodybuilding improves the physical appearance. |  |  |  |  |
| 14 | People who practice body building need a specific diet in comparison to those who do not practice it. |  |  |  |  |
| 15 | For a bodybuilder a specific diet balances the highest energy consumption (calories). |  |  |  |  |
| 16 | It is necessary to consult the doctor before taking food supplements. |  |  |  |  |
| 17 | It is necessary to consult the doctor before taking hormones . |  |  |  |  |
| 18 | It is safe to buy Food Supplements on websites . |  |  |  |  |
| 19 | It is safe to buy hormones on websites. |  |  |  |  |
| 20 | In order to improve sport performance, exceeding the recommended dose of a food supplement does not make a risk to the health. |  |  |  |  |
| 21 | To improve sport performance, would you take hazardous substances for the health (?). |  |  |  |  |
| 22 | To improve sport performance, would you recommend others to use Food Supplements (?). |  |  |  |  |
| 23 | To improve sport performance, would you recommend others to use Hormones(?). |  |  |  |  |
| 24 | Does the Gym provide exhaustive information about the use and types of food supplements(?). |  |  |  |  |
| 25 | You believe that the media provide complete information on the use and types of Food Supplements. |  |  |  |  |
| 26 | You believe that the labels of Food Supplements are detailed. |  |  |  |  |
| 27 | You believe that the laws on the use and the types of Food Supplements are exhaustive. |  |  |  |  |
| **BEHAVIOR** | | **Yes** | **Often** | **Sometimes** | **Never** |
| 28 | In order to compensate for the most energy expenditure and / or improve sports performance, do you follow a specific diet? |  |  |  |  |
| 29 | Do you take Food Supplements to offset the increased energy consumption and / or improve sports performance? |  |  |  |  |
| 30 | Do you take Hormones to offset the increased energy consumption and / or improve your sporting performance? |  |  |  |  |
| 31 | Have you ever exceeded recommended doses of Dietary Supplements to offset the increased energy consumption and / or improve sports performance. |  |  |  |  |
| 32 | Have you ever exceeded recommended doses of Hormones to offset the increased energy consumption and / or improve sports performance. |  |  |  |  |
| 33 | Advice to others the use of Dietary Supplements. |  |  |  |  |
| 34 | Advice to others the use of Hormones. |  |  |  |  |
| 35 | You buy Dietary Supplements on the Internet |  |  |  |  |
| 36 | You buy Dietary Supplements in the pharmacies. |  |  |  |  |
| 37 | You buy Hormones on the Internet. |  |  |  |  |
| 38 | You buy Hormones in the pharmacies. |  |  |  |  |
| 39 | When you buy Dietary Supplements you usually see the label. |  |  |  |  |
| 40 | You inform yourself at the gym about the use and type of Food Supplements to take. |  |  |  |  |
| 41 | You inform yourself at the gym about the use and type of Hormones to take. |  |  |  |  |
| 42 | Do you inform yourself on the Internet about the use and type of Dietary Supplements to take? |  |  |  |  |
| 43 | Do you inform yourself on the Internet about the use and type of Hormones to take? |  |  |  |  |
| 44 | Tell your doctor / nutritionist about the use and type of Food Supplements to be taken. |  |  |  |  |
| 45 | Tell your doctor / nutritionist about the use and type of hormones to be taken. |  |  |  |  |
| 46 | Do you update on the laws regulating the use and type of Dietary Supplements? |  |  |  |  |
| 47 | Do you update on the laws regulating the use and type of Hormones? |  |  |  |  |
